# SNPs across time and space: population genomic signatures of founder events and epizootics in the House Finch (*Haemorhous mexicanus*)

**DOI:** 10.1002/ece3.2444

**Published:** 2016-09-28

**Authors:** Allison J. Shultz, Allan J. Baker, Geoffrey E. Hill, Paul M. Nolan, Scott V. Edwards

**Affiliations:** ^1^ Department of Organismic and Evolutionary Biology and Museum of Comparative Zoology Harvard University Cambridge MA USA; ^2^ Department of Natural History, Royal Ontario Museum Department of Ecology and Evolutionary Biology University of Toronto Toronto ON Canada; ^3^ Department of Biological Sciences Auburn University Auburn AL USA; ^4^ Department of Biology The Citadel Charleston SC USA

**Keywords:** bottleneck, epizootic, founder effect, Hawaii, introduced populations, linkage disequilibrium, *Mycoplasma gallisepticum*, RADseq, selection

## Abstract

Identifying genomic signatures of natural selection can be challenging against a background of demographic changes such as bottlenecks and population expansions. Here, we disentangle the effects of demography from selection in the House Finch (*Haemorhous mexicanus*) using samples collected before and after a pathogen‐induced selection event. Using ddRADseq, we genotyped over 18,000 SNPs across the genome in native pre‐epizootic western US birds, introduced birds from Hawaii and the eastern United States, post‐epizootic eastern birds, and western birds sampled across a similar time span. We found 14% and 7% reductions in nucleotide diversity, respectively, in Hawaiian and pre‐epizootic eastern birds relative to pre‐epizootic western birds, as well as elevated levels of linkage disequilibrium and other signatures of founder events. Despite finding numerous significant frequency shifts (outlier loci) between pre‐epizootic native and introduced populations, we found no signal of reduced genetic diversity, elevated linkage disequilibrium, or outlier loci as a result of the epizootic. Simulations demonstrate that the proportion of outliers associated with founder events could be explained by genetic drift. This rare view of genetic evolution across time in an invasive species provides direct evidence that demographic shifts like founder events have genetic consequences more widespread across the genome than natural selection.

## 1 Introduction

Expansions of organisms into novel ranges or habitats are ubiquitous across the tree of life. All species experience range expansions and contractions through time, but human activities are accelerating the pace of range shifts through alteration of habitats and direct movement of species (Hulme, 2009). Introduced populations can have evolutionary impacts on native species in these new locations (Mooney & Cleland, 2001), but the introductions also have evolutionary consequences for the introduced populations themselves as they experience new environments and an altered demographic history (Baker & Moeed, 1987; Baker & Stebbins, 1965; Dlugosch & Parker, 2008). Introduced populations often experience founder effects and novel selection regimes (Dlugosch & Parker, 2008), and if the bottlenecks resulting from founder events are sufficiently long and severe, reduced genetic diversity and heterozygosity can be sustained even after subsequent population expansion (Nei, Maruyama, & Chakraborty, 1975). Although colonization events often begin with bottlenecks, they are frequently followed by rapid population expansions, a situation that can ameliorate long‐term reductions in genetic diversity and their detrimental effects (Dlugosch & Parker, 2008). On the one hand, these expansions can facilitate rapid morphological or physiological change (Reznick & Ghalambor, 2001) and the accumulation of deleterious mutations due to enhanced genetic drift on the edge of an expansion (Peischl, Dupanloup, Kirkpatrick, & Excoffier, 2013). Although fluctuations in population sizes are characteristic of invasive species, species in native ranges can also experience such changes in demography, especially when they are impacted by environmental alteration, habitat degradation and fragmentation, and climate change (Moran & Alexander, 2014; Wilcove, Rothstein, Dubow, Phillips, & Losos, 1998).

In addition to novel demographic shifts, introduced species can also encounter novel selection regimes in newly colonized habitats. Infectious diseases constitute one of the strongest selective pressures encountered in novel habitats and can have profound impacts on a species by increasing host mortality and decreasing reproductive output (Altizer, Harvell, & Friedle, 2003; Haldane, 1949; Karlsson, Kwiatkowski, & Sabeti, 2014). Modern global connectivity increases the rate at which organisms are exposed to novel pathogens to which they do not have previously‐evolved resistance (Daszak, Cunningham, & Hyatt, 2000). Emerging pathogens can have devastating effects on novel hosts, causing extinctions or severe population reductions (Dobson & Foufopoulos, 2001). Understanding the evolutionary dynamics between novel pathogens and hosts is of paramount importance to the preservation of biodiversity (Altizer et al., 2003), especially in the context of a demographic history reflecting past bottlenecks and range expansions. Additionally, the synergistic effects of population introductions and encounters with novel pathogens have rarely been studied (Longo, Burrowes, & Zamudio, 2014).

The House Finch (*Haemorhous mexicanus*), a common bird in both urban and rural environments in North America, has become a model for the study of adaptation to novel environments following introductions and for host‐pathogen coevolution (Badyaev et al., 2002; Bonneaud et al., 2011). The native range is confined to the western United States and Mexico, whereas the established populations in the eastern United States and Hawaii are the result of human‐mediated introductions in the 1940s and 1870s, respectively (Hill, 2002). Both populations were thought to have been introduced from a small number of founders (Elliott & Arbib, 1953; Grinnell, 1911), but underwent rapid population expansions and are abundant in their respective ranges (Hill, 2002), suggesting classic examples of bottlenecks followed by exponential population growth. Even though the Hawaiian and eastern US populations were recently derived, both populations exhibit morphological and behavioral differences from the founding populations (Able & Belthoff, 1998; Aldrich & Weske, 1978; Badyaev & Hill, 2000; Badyaev et al., 2002; Egbert & Belthoff, 2003; Vazquez‐Phillips, 1992), and genetic divergence has been detected with both mitochondrial DNA and multilocus datasets (Benner, 1991; Hawley, Briggs, Dhondt, & Lovette, 2008; Hawley, Hanley, Dhondt, & Lovette, 2006; Vazquez‐Phillips, 1992; Wang, Baker, Hill, & Edwards, 2003).

Despite extensive study, the nature and extent of genetic change in the House Finch as a result of human‐mediated introductions has been unclear, and only recently has it been suggested that different components of the House Finch genome may have responded to introductions in different ways (Backström, Shipilina, Blom, & Edwards, 2013; Hawley, DuRant, Wilson, Adelman, & Hopkins, 2012; Hawley et al., 2006, 2008; Hess, Wang, & Edwards, 2007; Vazquez‐Phillips, 1992; Wang et al., 2003; Zhang, Hill, Edwards, & Backström, 2014). A situation complicating the analysis of genetic diversity in the House Finch has been temporal evolution of populations as a result of a novel pathogen, *Mycoplasma gallisepticum* (MG), which precipitated an epizootic event that is now well documented by demographic and genetic studies as well as statistical models of host–pathogen coevolution (Dhondt et al., 2006; Staley & Bonneaud, 2015). After its first encounter with House Finches in the mid‐1990s in the mid‐Atlantic region, MG rapidly spread through the introduced eastern population, causing severe declines across the region as high as 60% in some areas (Dhondt, Tessaglia, & Slothower, 1998; Nolan, Hill, & Stoehr, 1998). MG reached native populations in the west in 2002, where it spread more slowly and with a lower prevalence (Dhondt et al., 2006). Both experimental and gene expression studies have revealed mounting evidence for genetic evolution in House Finches as a result of the MG epizootic (Adelman, Kirkpatrick, Grodio, & Hawley, 2013; Bonneaud, Balenger, Zhang, Edwards, & Hill, 2012; Bonneaud et al., 2011; Wang, Farmer, Hill, & Edwards, 2006), with the potential for reductions in genetic diversity as a result of the epizootic itself. Some studies surveying genetic diversity have been able to directly analyze House Finch populations sampled prior to the epizootic and thereby isolate human‐mediated introductions as a factor contributing to reductions in genetic diversity (Benner, 1991; Hawley & Fleischer, 2012; Hess et al., 2007; Vazquez‐Phillips, 1992; Wang et al., 2003). However, other studies with the aim of measuring changes in genetic diversity due to the human‐mediated introductions sampled populations after the epizootic, with the possibility that any reductions found may mistakenly be attributed to the introductions when in fact the consequences of the epizootic may have been at play (Backström et al., 2013; Hawley et al., 2006, 2008; Zhang, Hill et al., 2014).

**Figure 1:**
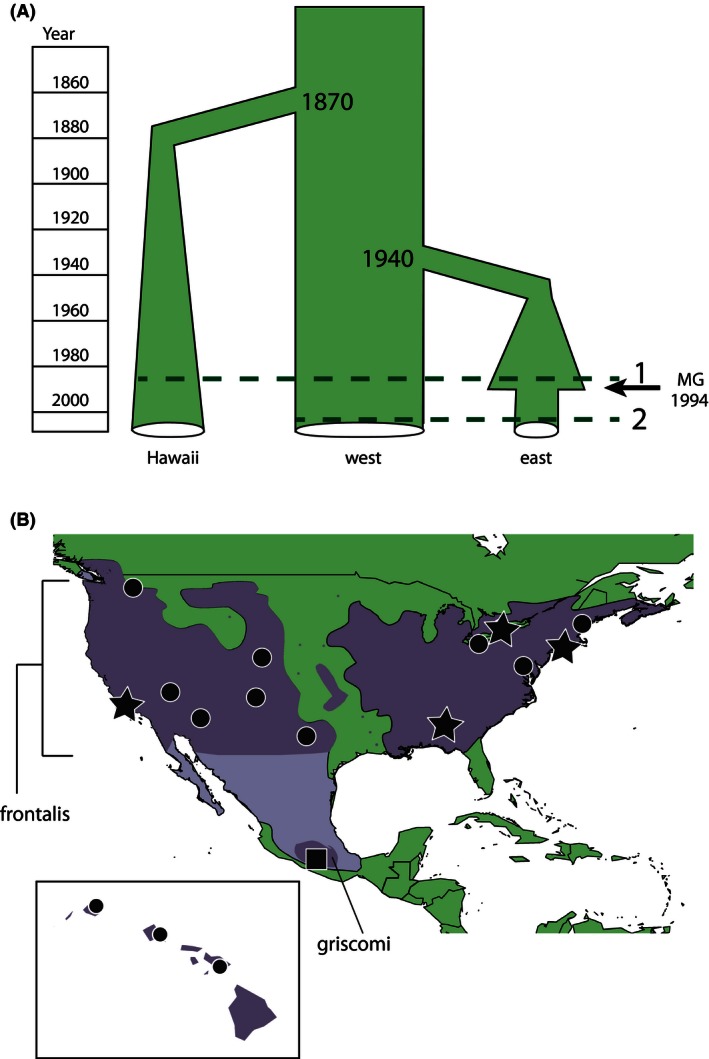
Approximate demographic history associated with population introductions in Hawaii and the eastern United States, and the MG epizootic event in the eastern United States. Line 1 on the figure indicates the approximate sampling time for the pre‐epizootic samples, and line 2 indicates the approximate sampling time for the post‐epizootic samples. (B) Map of the House Finch sampling localities, including Hawaii, and the range circa 1990 (NatureServe 2011; Ridgely et al. and Birdlife International 2011). All *frontalis* subpopulations (circles and stars) were sampled before the MG epizootic (line 1 on panel A), and the subpopulations indicated by a star were sampled again in 2001 or 2003, approximately eight generations after the MG epizootic (line 2 on panel A). The *griscomi* subspecies was also sampled (square). The ranges of the *frontalis* and *griscomi* subspecies are highlighted.

In this study, we compare the consequences for genetic diversity of both introductions and the epizootic by directly comparing geographically and temporally sampled populations of the House Finch. Demographic events, such as recent bottlenecks, confound the ability to study the genetic consequences of recent selection events (Domingues et al., 2012; Thornton, Jensen, Becquet, & Andolfatto, 2007). Genetic drift resulting from a bottleneck and selection event can each exhibit signatures of increased linkage disequilibrium and a reduction in effective population size, although demographic events typically have global effects on the genome, whereas the effects of selection events are genomically more local (Nielsen, 2005). By sampling the same populations before and after the MG epizootic (Fig. 1A), we have the rare opportunity to disentangle the signatures of drift and selection. Here, we use double‐digest restriction site associated sequencing (ddRADseq; Peterson, Weber, Kay, Fisher, & Hoekstra, 2012) to genotype thousands of markers across the House Finch genome and quantify the evolutionary history across all the chromosomes. RADseq and its variants have proved useful in phylogeographic studies as well as studies searching for *F*_ST_ outliers and other signatures of natural selection in populations with the complex recent histories (Andrews, Good, Miller, Luikart, & Hohenlowe, 2016; Edwards, Shultz, & Campbell‐Staton, 2016; Hohenlohe, Bassham, Currey, & Cresko, 2012; Hohenlohe et al., 2010; Ruegg, Anderson, Boone, Pouls, & Smith, 2014). With our combination of temporal sampling and genome‐scale genotyping, we provide a comprehensive picture of the population genetic history of this emerging model system.

## 2 Materials and Methods

### 2.1 Sampling

To quantify the effects of the introductions on genetic diversity, we examined individuals collected before reports of the MG epizootic in each region. Summaries of the sampling can be found in Table 1, and the collection number, collection dates, and specific localities of each specimen can be found in Table S1. Following a strategy similar to that of Wang et al. (2003), we sampled 16 pre‐epizootic populations of the *frontalis* subspecies of House Finch in North America and the Hawaiian Islands (circles and stars, Fig. 1B; Table 1), for a total of 90 individuals. To examine the effects of the epizootic, we analyzed samples from three of the eastern subpopulations in 2001 or 2003, approximately eight generations after the MG epizootic (*n* = 18; stars, Fig. 1B; Table 1). We also analyzed samples from one western subpopulation from California in 2003, before the epizootic in this region, but across the same time span as in the eastern United States, allowing us to control for population genetic differences occurring in House Finches during this time span but not due to the epizootic (throughout the study, we will refer to pre‐ and post‐eastern and western populations as Pre‐E, Post‐E, Pre‐W, and Post‐W respectively). To identify derived alleles within the *frontalis* subspecies, we also sampled individuals from the *griscomi* (*n* = 6) subspecies (square, Fig. 1B; Table 1) and two closely related sister species as outgroups, the Purple Finch (*H. purpurus*;* n* = 3) and the Cassin's Finch (*H. cassinii*;* n* = 3) (Smith, Bryson, Chua, Africa, & Klicka, 2013; Table 1). Blood samples were preserved in Queen's lysis buffer (Seutin, White, & Boag, 1991) and stored at −80**°**C. Tissue samples were frozen in liquid nitrogen following collection, and stored at −80**°**C, and transferred between laboratories in 100% ethanol at room temperature.

**Table 1:** Population sample information

Regional population	Locality	Pre‐ or post‐epizootic	Tissue type	No. of samples sequenced	No. of samples analyzed
Western	Arizona (AZ)	Pre	Tissue	6	6
	California (CA)	Pre	Tissue	6	6
		Post	Blood	6	6
	Colorado (CO)	Pre	Tissue	6	6
	New Mexico (NM)	Pre	Tissue	6	6
	Nevada (NV)	Pre	Tissue	6	6
	Texas (TX)	Pre	Tissue	6	6
	Washington (WA)	Pre	Tissue	6	5
	western totals	Pre		42	41
		Post		6	6
Eastern	Alabama (AL)	Pre	Blood	6	6
		Post	Blood	6	6
	Maine (ME)	Pre	Tissue	6	6
	New York (NY)	Pre	Tissue	6	6
		Post	Blood	6	4
	Ohio (OH)	Pre	Tissue	6	6
	Ontario (ON)	Pre	Tissue	6	6
		Post	Blood	6	5
	eastern totals	Pre		30	30
		Post		18	15
Hawaiian	Kauai (HK)	NA	Tissue	6	3
	Maui (HM)	NA	Tissue	6	3
	Oahu (HO)	NA	Tissue	6	6
	Hawaiian totals	NA		18	12
*H. m. frontalis totals*		Pre		90	83
		Post		24	21
Outgroup	Guerrero, Mexico (*griscomi*) (GU)	NA	Tissue	6	5
	*Haemorhous cassinii* (CC)	NA	Tissue	3	2
	*Haemorhous purpeurus* (CP)	NA	Tissue	3	2

### 2.2 RAD sequencing

We extracted whole genomic DNA using the DNeasy Blood and Tissue Kit (Qiagen Inc., Valencia, CA), using the standard blood and tissue protocols as appropriate. Each individual was barcoded and genotyped following a ddRADseq (Peterson et al., 2012) protocol using the SphI‐EcoR1 enzyme combination and isolating fragments in the range of 345–407 base pairs (bp). Details of the protocol can be found in the supplemental methods. We sequenced our library at the Bauer Core Facility of the FAS Center for Systems Biology at Harvard University (Cambridge, MA), using one lane of an Illumina HiSeq 2500 Rapid Run flow cell with 150 base pair paired‐end sequencing.

### 2.3 Computational analysis and bioinformatics

Sequence data were demultiplexed using Geneious version 6.0.5 (Kearse et al., 2012) allowing for a single mismatch in the barcode. We then trimmed the four base pair restriction sites and used the *process_radtags.pl* program from STACKS version 0.99994 (Catchen, Amores, Hohenlohe, Cresko, & Postlethwait, 2011; Catchen, Hohenlohe, Bassham, Amores, & Cresko, 2013) to filter low‐quality reads (on average, ~6% of reads were discarded per individual due to low‐quality scores). We employed a *de novo* approach to build a catalog of loci and call SNPs. Of the 126 individuals sequenced, we removed 13 that had fewer than 100,000 reads from downstream analyses (See Table 1 for final numbers of individuals analyzed per subpopulation). Preliminary analyses confirmed that these individuals had very low coverage and contributed very few loci to downstream analyses.

### 2.4 De novo assembly

We used STACKS version 1.21 to create a *de novo* catalog of loci and call SNPs (Catchen et al., 2011, 2013). We merged all reads into a single file for *de novo* locus identification and trimmed them to a length of 140 base pairs. The optimal set of parameters for library assembly varies according to study system (Catchen et al., 2011, 2013; Mastretta‐Yanes et al., 2014), so with the pre‐epizootic set of individuals, we tested a range of parameters with the *denovo_map.pl* pipeline for catalog construction. By creating a catalog of loci with both reads simultaneously, we were able to leverage information about which reads were derived from the same DNA fragments, which in turn allowed us to validate and test parameter performance. Briefly, we tested values of –*M* and –*n* from 1 to 8 and –*m* with 4, 10, and 20. We assessed performance based on the number of loci in the final dataset, the percentage of loci from the same DNA fragment that had more than a one‐to‐one match within an individual, and the percentage of loci that had more than two alleles within an individual. Final parameters used for downstream analysis were –*M* 4, –*n* 4, and –*m* 4. Details on parameter testing and novel python scripts are available in the supplemental material.

With the *populations* program in STACKS, we generated three different datasets for subsequent analyses. The first two datasets included all individuals (including both pre‐epizootic and post‐epizootic time periods), but differed in SNP filtering. Dataset 1 used a less conservative SNP filter, requiring an individual minimum locus depth of 10 to be included (–*m* 10), and a locus to only be included if it was present in at least 75% of individuals in half of the populations (–*r* .75 and –p 11). The second, more conservative filter (dataset 2) required an individual minimum locus depth of 30 (–*m* 30) and had the same inclusion parameters. Analyses of dataset 2 produced results very similar to those of dataset 1, although sometimes less resolved due to the smaller number of loci, so we only present the results of dataset 1 throughout the rest of the article. Individuals from all time periods were used to build these STACKS catalogs. We also ran some preliminary analyses on datasets with more stringent completeness filters, but found that analyzing these datasets had very little effect on overall results. The final dataset (dataset 3) focused on maximizing high‐quality SNPs from the temporal samples for analyses seeking to identify MG‐mediated selection. This dataset only included individuals from the diachronically sampled subpopulations (CA, AL, NY, and ON), but included both time periods. The quality filter required an individual minimum locus depth of 30 (–*m* 30), and a locus was to be included if it was present in at least 75% of individuals in two of the samples: Pre‐E, Post‐E, Pre‐W, and Post‐W (–*r* .75 –p 2).

We further refined each dataset by removing possible problematic loci that matched any of the following three criteria: the locus contained a SNP with observed heterozygosity greater than 0.75; the locus contained a SNP that was not in Hardy–Weinberg equilibrium (*p* < .05) in at least two of the three populations (eastern, western, and Hawaiian); or a locus mapped to the Zebra Finch (*Taeniopygia guttata*) genome version 3.2.4 (Warren et al., 2010) with BLASTN 2.2.29+ (Camacho et al., 2009) more than once with an e‐value less than 10^−40^. The Zebra Finch is the closest relative to the House Finch with a high‐quality reference genome. The House Finch and Zebra Finch lineages diverged approximately 50 million years ago (Brown, Rest, Garcia‐Moreno, Sorenson, & Mindell, 2008), and given the conservatism of the avian genome (Ellegren, 2013; Zhang, Li et al., 2014), the Zebra Finch genome has successfully been used to map RADseq reads from other similarly diverged bird species (Bourgeois et al., 2013). Finally, we sought to remove any related individuals from the dataset. We used the program KING (Manichaikul et al., 2010) to estimate relatedness among all individuals. We identified two individuals in the post‐epizootic sample from Ontario that displayed a kinship value of 0.1534, indicative of a second‐degree relationship, so we removed one of the individuals, indiv_121, from all downstream analyses. All datasets are available in the Dryad repository (http://dx.doi.org/10.5061/dryad.0h2g0).

### 2.5 Population structure

To test for population structure among pre‐epizootic subpopulations, we used a Bayesian approach, implemented in the program STRUCTURE 2.3.4 (Pritchard, Stephens, & Donnelly, 2000), to determine the number of genetic groups or clusters (*K*) that best fit the data and to assign individuals to these clusters. We first tested for structure among all pre‐epizootic individuals, including outgroups (*n* = 92 individuals). Because variation among higher levels of population structure can mask substructuring (Evanno, Regnaut, & Goudet, 2005), we implemented a hierarchical set of analyses. We tested for structure within the *frontalis* subspecies (including the introduced populations; *n* = 83 individuals); within the native *frontalis* population (“western” population; *n* = 41 individuals); within the introduced eastern *frontalis* population (“eastern” population; *n* = 30); and within the introduced Hawaiian *frontalis* population (“Hawaiian” population; *n* = 12). To determine the optimal number of clusters (*K*), we considered the highest Δ*K*, as recommended by Evanno et al. (2005), but also considered the biological feasibility of the result. For all analyses, we used a SNP dataset that only contained a single SNP per locus, and we ran four replicates of each *K* value ranging from 1 to 8 with an admixture model, burn‐in of 100,000, and 1,000,000 Markov chain Monte Carlo samples. We used STRUCTURE HARVESTER (Earl & vonHoldt, 2011), CLUMPP v. 1.1.2 (Jakobsson & Rosenberg, 2007), and DISTRUCT v. 1.1 (Rosenberg, 2004) to visualize the combined results.

### 2.6 Population genetic analyses

We used the *populations* program in STACKS (Catchen et al., 2011, 2013) to calculate summary statistics within each subpopulation in both time periods when possible. We also calculated summary statistics for all pre‐epizootic individuals grouped by population (western, eastern, and Hawaiian). We further assessed population differentiation by using the *populations* program to calculate *F*_ST_ for each nucleotide present in each pair of subpopulations. We calculated Tajima's *D* for each subpopulation and assessed a significant difference from the neutral expectation using the beta distribution (Tajima, 1989; python script available at https://github.com/ajshultz/Rad/).

We calculated linkage disequilibrium (LD; *r* ^2^; Hill & Robertson, 1968) to compare the levels of nonindependence of SNPs among the Pre‐E, Pre‐W, Hawaiian, and Post‐E populations using a custom python script (*Pairwise_linkage_disequilibrium.py* available at https://github.com/ajshultz/Rad/). Because sample size can affect measures of LD, for each population we randomly chose eight individuals with less than 50% missing data. First, we calculated *r* ^2^ between all pairs of SNPs located on the same locus (between 1 and 139 base pairs apart). Second, we compared the mean *r* ^2^ value between SNPs located on read 1 and read 2 loci from the same DNA fragment (paired via *catalog_read_pair.py*, described in the supplemental methods). We compared these values to the equivocal number of randomly chosen pairs from the catalog of possible loci. By comparing levels of LD from loci on the same DNA fragment to levels from randomly chosen loci, we could assess whether the decrease in LD observed in the single locus analysis degraded to a level of LD lower than that observed in a single read. Additionally, by confirming that levels of LD were similar among populations with randomly chosen loci, we could ensure that any significant differences observed in loci in a single read we observed among populations were not an artifact of dataset structure (e.g., differences among populations in the amount of missing data).

### 2.7 Selection scans

We used two methods to test for selection between the native and introduced populations, using Pre‐W versus Pre‐E; Pre‐W versus Hawaiian with dataset 1; Pre‐E versus Post‐E (combining AL, ON, and NY) samples with datasets 1 and 3; and Pre‐W versus Post‐W (CA) samples with datasets 1 and 3. First, we used the *populations* program in STACKS to calculate allele frequency differences for each SNP found in both populations. We assessed the significance for these differences with a Fisher's exact test, corrected for multiple testing to a 5% false discovery rate using the Benjamini–Hochberg approach (Benjamini & Hochberg, 1995). This approach for detecting allele frequency shifts has advantages compared to using FST outliers, which can be influenced by levels of within‐population heterozygosity (Cruickshank & Hahn, 2014). Second, we used BayeScan version 2.1 (Foll & Gaggiotti, 2008) for each of the same population comparisons separately with a burn‐in of 50,000 iterations and 100,000 generations of data collection. Because BayeScan can be sensitive to loci with low minor allele frequencies, we first filtered each dataset to include only loci with a minor allele frequency greater than 0.10.

We simulated datasets to generate expectations for the proportion of outlier loci under the neutral model for an introduction event. We used the program ms (Hudson, 2002) to generate datasets that approximated historical records and varied the size of the introduced founding population (Fig. S1; supplemental methods) to explore the effect the bottleneck size had on the proportion of outliers. We ran the model using a mutation rate estimated for Zebra Finch of 2.21 × 10^−9^ per site per year (Nam et al., 2010) and a generation time of 1 year. We simulated 1,000 datasets for each founding *N*_e_ (20, 200, 2,000, 100,000, no bottleneck) twice, once with sampling modeled after the Pre‐W versus Pre‐E comparisons (82 and 60 chromosomes, respectively) and once with sampling modeled after the Pre‐W versus Hawaiian comparisons (82 and 24 chromosomes, respectively). For all simulations, we calculated the significance of allele frequency differences between populations with a Fisher's exact test, corrected for multiple testing as described above.

## 3 Results

### 3.1 Sequencing and de novo library construction

We obtained a total of 40,151,299 paired‐end 150 base pair reads that passed Illumina's quality filter for 126 individuals (mean 637,322 total reads per individual; Table S1). The *de novo* assembly generated an average of 12,700 unique loci per individual, of which an average of 3,217 were polymorphic (Table S1). Across the three datasets, when loci were mapped to the Zebra Finch genome with a minimum e‐value of 10^−40^, 12% of loci consistently mapped more than once and were subsequently dropped from the analysis. Approximately 65% of loci mapped just once to the Zebra Finch genome, and 24% did not map at all. Loci fell evenly across the entire genome (Fig. S2), and the number of loci per chromosome was significantly correlated with Zebra Finch chromosome length (dataset 1: *R* ^2^ = 94%, *p* < .0001; Fig. S3). After filtering for multiple hits to the Zebra Finch genome and deviations from Hardy–Weinberg equilibrium, dataset 1 (with all individuals and a minimum locus depth of 10) contained 2,283 loci and 18,096 SNPs, was 73% complete, and had a mean depth per locus of ~60. Dataset 2 (all individuals and a minimum locus depth of 30) contained 889 loci and 6,877 SNPs, was 67% complete, and had a mean depth of ~68. Dataset 3 (only diachronically sampled populations and a minimum stack depth of 30) contained 2,150 loci and 8,561 SNPs, was 55% complete, and had a mean depth of ~66. Across all datasets, there was a high amount of variability in the amount of data missing in any particular individual (Fig. S4), most likely caused by sensitivities of the ddRADseq protocol to tissue degradation and long‐term storage.

### 3.2 Population structure

**Figure 2:**
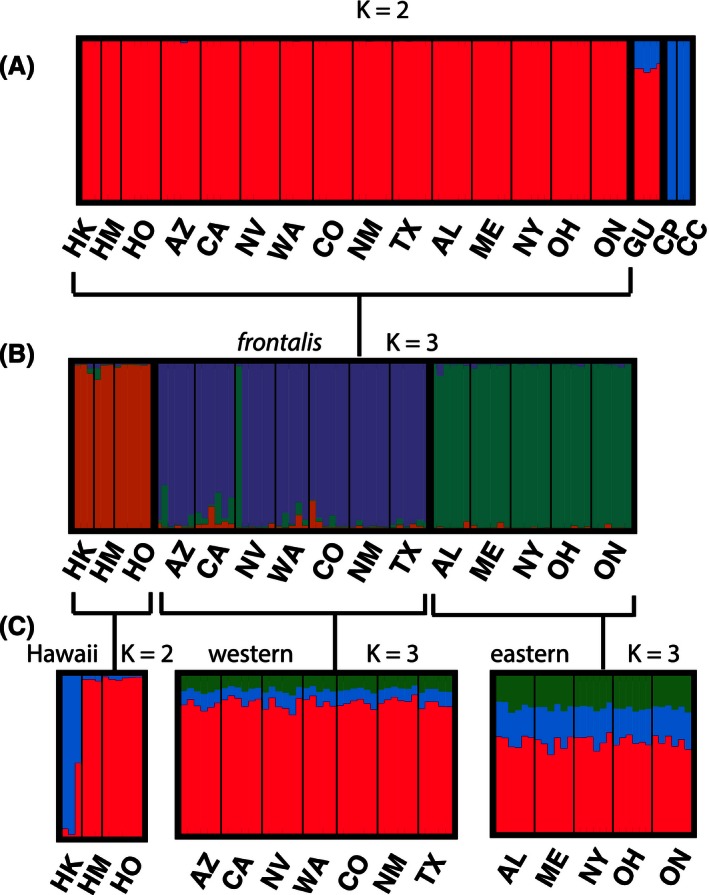
(A) STRUCTURE plot with *K* = 2 for all pre‐epizootic individuals using dataset 1. Abbreviations for populations indicated in STRUCTURE plots are as follows: HK = Kauai, HM = Maui, HO = Oahu, AZ = Arizona, CA = California, NV = Nevada, WA = Washington, CO = Colorado, NM = New Mexico, TX = Texas, AL = Alabama, ME = Maine, NY = New York, OH = Ohio, ON = Ontario, GU = Guerrero, CP = Purple Finch, CC = Cassin's Finch. (B) STRUCTURE plot with *K* = 3 for all *frontalis* individuals. (C) STRUCTURE plot with the *K* = 2 for all Hawaiian individuals, *K* = 3 for western individuals, and *K* = 3 for eastern individuals

When comparing all individuals across all three species sampled, the Evanno method (Evanno et al., 2005) identified two clusters as optimal, separating the outgroup species from the House Finch (Fig. 2A; Table S2). Among only the *frontalis* House Finch birds, *K* = 3 was optimal (Table S2), separating out the native western, introduced Hawaiian, and introduced eastern populations (Fig. 2B). Among the Hawaiian birds, *K* = 2 was optimal (Table S2), which separated the Kauai individuals from those sampled on Oahu and Maui (Fig. 2C). For both the eastern and western populations considered alone, *K* = 3 was optimal (Table S2), but this result did not appear to contain any biologically useful information, with three clusters equally likely in all individuals (Fig. 2C). The Evanno method cannot identify *K* = 1 as the optimal strategy (Evanno et al., 2005), but that is likely the true model in this situation.

### 3.3 Population genetics

**Figure 3:**
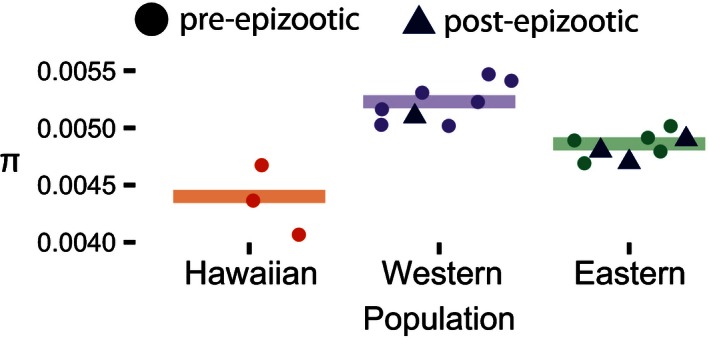
Estimates of diversity (π) for all subpopulations from dataset 1. The bars indicate the mean pre‐epizootic π value. Reductions in nucleotide diversity (π) are more severe in the Hawaiian population than the eastern population, with 15.8% and 7.0% reductions in the estimated mean subpopulation π, respectively. Pre‐epizootic estimates are circles, and post‐epizootic estimates are triangles

Using all 18,096 SNPs in dataset 1, the Pre‐W population had consistently higher levels of polymorphic sites, private sites, observed heterozygosity, and nucleotide diversity than the Pre‐E population or Hawaiian population (Table 2). These results hold true when considering subpopulations individually as well. Reductions in nucleotide diversity (π) relative to the western region were more severe for Hawaiian birds (−14%) than for eastern birds (−7%; Fig. 3) and were similar for haplotype diversity and heterozygosity (Table 2). Levels of diversity among Pre‐ and Post‐E birds were similar. Although the small number of populations sampled in both time periods makes statistical comparison difficult, Post‐E estimates were within two standard deviations of the Pre‐E range for both π and observed heterozygosity (Table 2; Fig. 3). We further confirmed that our results were qualitatively similar and not a by‐product of sample size by examining the results with eight individuals chosen randomly from each population (Pre‐E, Pre‐W, Hawaiian; results not shown). Tajima's *D* was negative, but nonsignificant for all populations, except for the post‐epizootic New York population. We found similar results for the dataset with eight randomly chosen individuals per population to counter the effects of different sample sizes (Pre‐W = −0.34, Pre‐E = −0.36, Post‐E = −0.36, Hawaiian = −0.38; all *p* > .05).

**Table 2:** Summary statistics for all populations calculated for dataset 1

Regional population	Locality	Pre‐ or post‐epizootic	Mean N per locus	Private alleles	Total sites across dataset	Polymorphic sites	% Polymorphic loci	Mean freq. of major allele (P)	Observed heterozygosity	Nucleotide diversity (π)	Haplotypediversity (h)
Western	Regional	Pre	34.42	3,354	309,312	11,585	3.75	.9965	0.0046	0.0054	0.78
	Arizona (AZ)	Pre	5.83	551	300,467	5,401	1.80	.9967	0.0047	0.0054	0.77
	California (CA)	Pre	5.60	286	242,436	4,024	1.66	.9969	0.0044	0.0050	0.71
		Post	5.68	354	242,420	4,094	1.69	.9968	0.0045	0.0051	0.72
	Colorado (CO)	Pre	5.61	351	236,289	4,063	1.72	.9968	0.0046	0.0052	0.74
	New Mexico (NM)	Pre	5.67	251	157,265	2,728	1.73	.9967	0.0047	0.0053	0.76
	Nevada (NV)	Pre	5.66	289	196,967	3,520	1.79	.9966	0.0048	0.0055	0.80
	Texas (TX)	Pre	5.35	260	179,373	2,972	1.66	.9968	0.0046	0.0052	0.73
	Washington (WA)	Pre	4.70	340	294,586	4,465	1.52	.9969	0.0044	0.0050	0.73
Eastern	Regional	Pre	26.11	384	309,404	7,591	2.45	.9967	0.0045	0.0051	0.72
	Alabama (AL)	Pre	5.80	79	299,625	4,636	1.55	.9969	0.0044	0.0049	0.68
		Post	5.75	90	312,905	4,781	1.53	.9969	0.0043	0.0049	0.68
	Maine (ME)	Pre	5.58	59	267,323	3,995	1.49	.9969	0.0043	0.0048	0.70
	New York (NY)	Pre	5.48	44	239,907	3,614	1.51	.9969	0.0046	0.0049	0.70
		Post	3.52	47	254,607	3,046	1.20	.9971	0.0042	0.0047	0.68
	Ohio (OH)	Pre	5.67	35	190,969	2,955	1.55	.9968	0.0046	0.0050	0.71
	Ontario (ON)	Pre	5.52	70	287,609	4,202	1.46	.9970	0.0042	0.0047	0.67
		Post	4.80	61	284,382	3,959	1.39	.9970	0.0043	0.0048	0.68
Hawaiian	Regional	NA	10.45	393	309,429	5,373	1.74	.9969	0.0041	0.0047	0.67
	Kauai (HK)	NA	3.00	103	287,622	2,654	0.92	.9974	0.0039	0.0041	0.57
	Maui (HM)	NA	3.00	35	114,494	1,191	1.04	.9971	0.0044	0.0047	0.70
	Oahu (HO)	NA	5.69	208	293,342	3,826	1.30	.9972	0.0039	0.0044	0.62
Outgroup	Guerrero, Mexico (*griscomi*) (GU)	NA	4.48	959	272,498	2,815	1.03	.9975	0.0035	0.0038	0.56
	*Haemorhous cassinii* (CC)	NA	2.00	1,417	129,397	1,172	0.91	.9971	0.0044	0.0049	0.71
	*Haemorhous purpeurus* (CP)	NA	2.00	1,588	143,094	1,153	0.81	.9973	0.0029	0.0045	0.65

Average pairwise *F*_ST_ among pre‐epizootic populations were consistent with the STRUCTURE analyses. For dataset 1, the species outgroups were the most divergent from the *frontalis* individuals (0.404–0.555, mean = 0.455; Fig. S5), followed by the subspecies outgroup (0.128–0.213; mean = 0.152). Populations within the eastern and western regions were the least differentiated (0.049–0.064, mean = 0.056), whereas levels of differentiation between eastern and western subpopulations were slightly higher (0.061–0.070, mean = 0.065). The Hawaiian population was the most differentiated from the other *frontalis* populations (0.071–0.102, mean = 0.086); *F*_ST_ was even higher between Kauai and the other two islands within Hawaii (0.099–0.129).

**Figure 4:**
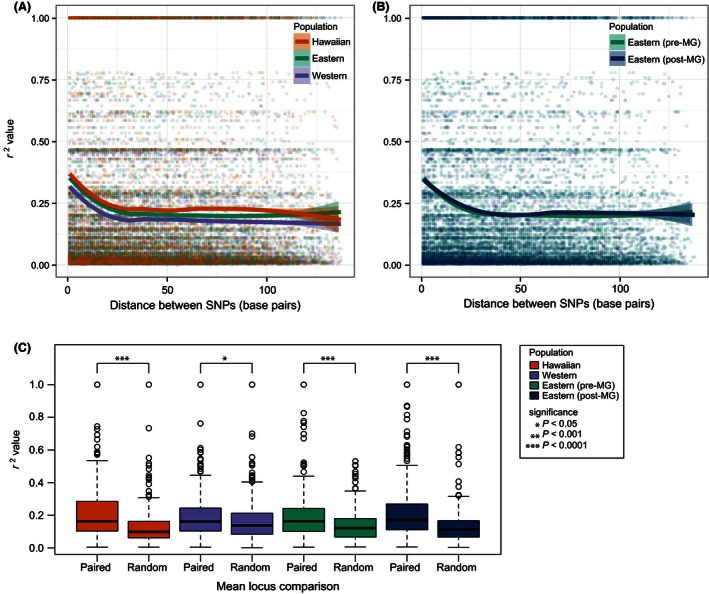
(A) Pre‐epizootic population linkage disequilibrium (*r* ^2^) calculations between the pairs of SNPs located on the same RAD locus. From eight randomly chosen individuals with less than 50% missing data, the LOESS smoothed *r* ^2^ values (solid lines) and 95% SE confidence intervals (shaded areas) are shown for each SNP distance. (B) LD (*r* ^2^) calculations between pairs of SNPs located on the same RAD locus. For eight Pre‐E and Post‐E individuals, the LOESS smoothed *r* ^2^ values (solid lines) and 95% SE confidence intervals (shaded areas) are shown for each SNP distance. (C) Mean LD (*r* ^2^) between SNPs on read 1 and read 2 of the same DNA fragment (paired; 375 pairs of loci) compared to two randomly chosen loci (random; 375 random pairs)

The Pre‐W population exhibited lower levels of LD than either the Pre‐E or Hawaiian populations (Fig. 4A; Pre‐W vs. Pre‐E Wilcoxon rank‐sum test *p* < .0001, Pre‐W vs. Hawaiian Wilcoxon rank‐sum test *p* < .0001), but we found no difference in LD between Pre‐ and Post‐E populations (Fig. 4B; Wilcoxon rank‐sum test *p* = .282). There were 375 loci from read 1 and read 2 of the same DNA fragment as calculated by our novel python script (Supplemental methods). Levels of LD were higher for SNPs on pairs of loci from the same DNA fragment than for SNPs on randomly chosen pairs for all populations (Fig. 4C; Wilcoxon rank‐sum tests: Pre‐W, *p* = .0119, Hawaiian, *p* < .0001, Pre‐E, *p* < .0001, Post‐E, *p* < .0001). However, there were no differences among paired locus *r* ^2^ values from different populations (Wilcoxon rank‐sum test: Pre‐W vs. Pre‐E, *p* = .827; Pre‐W vs. Hawaiian, *p* = .361; Pre‐E vs. Post‐E, *p* = .160).

### 3.4 Selection scans

**Figure 5:**
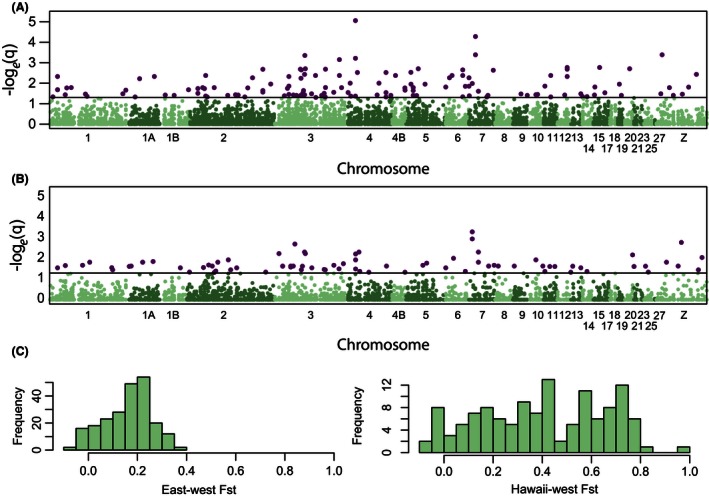
False discovery rate‐corrected q‐value from Fisher's exact test for different allele frequencies between (A) Pre‐E and Pre‐W populations and (B) Hawaiian and Pre‐W populations plotted according to position on the Zebra Finch genome. A horizontal black line indicates the 5% significance threshold, and the SNPs with q‐values that fall below this threshold used in downstream analyses are colored purple. (C) Distribution of *F*_ST_ values observed in outlier loci identified in (A) and (B) for Pre‐E and Pre‐W and Hawaiian and Pre‐W comparisons

Using dataset 1, we detected 224 SNPs whose frequencies were significantly different between the Pre‐E and Pre‐W populations (out of 12,928 comparisons; 1.7% of SNPs) with a FDR of 5%. Of these, 136 could be mapped to the Zebra Finch genome (Fig. 5A; Table S3). We detected 125 SNPs whose frequencies were significantly different between the Hawaiian and Pre‐W populations (out of 12,390 comparisons; 1.0% of SNPs) with a FDR of 5%. Of these, 84 could be mapped to the Zebra Finch genome (Fig. 5B; Table S4). Outlier loci from the Pre‐E versus Pre‐W comparison had a smaller range of *F*_ST_ values compared to Hawaiian and Pre‐W comparisons (Fig. 5C), with little overlap of loci identified as outliers in the two comparisons (Fig. S6). There were no SNPs significantly differentiated in the Pre‐ and Post‐E comparisons, and only three SNPs were significantly differentiated in the Pre‐W and Post‐W comparisons, none of which mapped to the Zebra Finch genome.

BayeScan detected four outliers in the Pre‐E/W comparison with a FDR of 5%, three of which could be mapped to the Zebra Finch genome (Locus 761, SNP 20; Locus 1261, SNP 43; Locus 1538, SNP 138; Table S3). BayeScan detected four outliers from two loci in the Hawaiian versus Pre‐W comparison, of which one was mappable (Locus 5849, SNP 55; Table S4). All loci identified by BayeScan were also detected in outlier analyses reported above.

Simulations of founding events under the neutral model had proportions of significant allele frequencies greater than or comparable to those found in our empirical data (Table 3). This holds true across a range of bottleneck sizes for both eastern and Hawaiian outlier loci.

**Table 3:** Proportion of outlier loci found in founder event simulations under the neutral model. We report the proportion of loci found with significant allele frequency differences (Fisher's exact test *p* < .05 after multiple test correction) from simulations of the founder event with five different founding population sizes. We simulated data 1,000 times for each founding population size with samples sizes that matched both eastern–western comparisons and Hawaii–western comparisons. We report the mean of each set of replicates ± standard error, and the empirical proportion calculated from dataset 1

Founder *N*_e_	Eastern sample size	Hawaiian sample size
20	0.343 ± 0.006	0.209 ± 0.005
200	0.042 ± 0.003	0.028 ± 0.002
2,000	0.010 ± 0.002	0.010 ± 0.001
100,000	0.006 ± 0.001	0.010 ± 0.001
No Bottleneck	0.005 ± 0.001	0.010 ± 0.001
Empirical	0.017	0.010

## 4 Discussion

Both natural selection and drift due to founder events can lead to genotypic changes in populations, and disentangling the relative contributions of these evolutionary forces can be difficult if one relies solely on contemporary samples from a single time period. Comparisons of the genotypes of populations across time and space enable better deductions of the evolutionary processes that underlie genomic change (Mathieson et al., 2015). To understand the relative importance to House Finch evolution of founder events and introduction to a novel habitat versus natural selection imposed by a novel pathogen, we employed ddRADseq to conduct a genome‐wide survey of genetic variation in introduced and native populations of the House Finch both before and after an epizootic event. In addition to providing insight and clarification of conflicting results from previous studies on the genetic effects of introductions in the eastern United States and Hawaii, ours is an early study using genome‐wide SNPs to quantify the effects of a major selection event with temporal sampling (see also Tin, Arora, Seeley, & Mikheyev, 2015).

### 4.1 Population genetic signatures of introduced populations

Our study suggests that ddRADseq data have great power to detect even subtle changes in effective population size and are well suited for population and conservation genomic studies even if a reference genome is not available (e.g., Backström, Qvarnstrom, Gustafsson, & Ellegren, 2006; Stapley, Birkhead, Burke, & Slate, 2008; Dierickx, Shultz, Sato, Hiraoka, & Edwards, 2015; Edwards, Shultz, & Campbell‐Staton, 2016). We demonstrate that despite the brevity of the bottlenecks prior to population expansions, the introduced populations of House Finches in the eastern United States and Hawaii have reduced genetic and haplotype diversity and heterozygosity. Although the number of individuals sampled in subpopulations was small compared to historical studies using one or a few loci, the large number of markers gives robust estimates of genetic diversity (Carling & Brumfield, 2007; Felsenstein, 2006; Mccormack, Hird, Zellmer, Carstens, & Brumfield, 2011; Therkildsen et al., 2013), and the concordance among estimates within geographic regions and subsamples (not shown) lends confidence to our results. Finally, despite biases that can be associated with ascertainment of ddRADseq data (Arnold, Corbett‐Detig, Hartl, & Bomblies, 2013), our estimates of genetic variability in the House Finch genome are similar to other species that survey noncoding genomic regions via next‐generation approaches, including species with both higher and lower estimates (Table S6). Although we corroborated several patterns of genetic differentiation found in previous work, including genetic differentiation of introduced House Finch populations, and no population differentiation within either western or eastern populations (Fig. 2; Wang et al., 2003; Hawley et al., 2006, 2008), for the first time, we show that genetic structure exists among birds from the Hawaiian Islands, with birds from Kauai, the most geographically isolated Hawaiian Island (Roderick & Gillespie, 1998), showing differentiation from those from Oahu and Maui. One individual from the Nevada population was consistently assigned to the eastern population with a very high proportion of its genome (98%). This individual could be an example of a rare migrant from the eastern population to the western population, but without re‐extracting and genotyping this individual, we cannot rule out the possibility of a mislabeled or misidentified sample.

We also used LD to study the dynamics of introductions in the House Finch. We quantified *r* ^2^ both between SNPs within single 140‐bp reads and between SNPs on loci from paired ends of ~300 base pair fragments. We confirm the rapid LD decay in very short segments of the genome observed in a few candidate loci sequenced by Backström et al. (2013), as well as in natural populations of other birds, at least for autosomes (Balakrishnan & Edwards, 2008; Edwards & Dillon, 2004; Kawakami et al., 2014). We also demonstrate elevated genome‐wide levels of LD in introduced populations, a key prediction of bottleneck scenarios (Slatkin, 2008). Balakrishnan and Edwards (2008) detected elevated LD in an island population of Zebra Finches, which was accompanied by a tenfold decrease in nucleotide diversity in the island population. Our results suggest that elevated LD can persist even after a rapid expansion event and more modest decreases in genetic diversity.

### 4.2 Selection versus drift during human‐induced introductions

Regions of the genome exhibiting significant differentiation as a result of human introductions could be due either to adaptation to the novel environment or to the genetic effects of a bottleneck (Lee, 2002; Thornton, Jensen, Becquet, & Andolfatto, 2007; Poh, Domingues, Hoekstra, & Jensen, 2014). Despite the low LD and sparse genome sampling afforded by ddRADseq, we detected 224 SNPs with significant allele frequency differences between the pre‐epizootic eastern and western populations and 125 SNPs with significant allele frequency differences between the Hawaiian and pre‐epizootic western populations. Some estimates of allele divergence, such as *F*_ST_, are influenced by underlying levels of polymorphism (Cruickshank & Hahn, 2014). However, by focusing on loci with significant measures of allele differentiation via a Fisher's exact test, rather than by diversity‐dependent measures such as *F*_ST_, we avoid these biases. Indeed, the power to detect allelic differentiation in our dataset appears substantial only in regions of the genome with adequate polymorphism; Fig. S7 suggests that only genomic regions with moderate levels of diversity yielded higher or outlier values of *F*_ST_. Our simulations suggest that a large proportion of these shifts in allele frequency differences are likely a result of genetic drift during the bottleneck event (Thornton et al., 2007) or allele surfing during the population expansions (Excoffier & Ray, 2008). Genetic drift can increase the variance in allele frequencies in small populations (Nei & Tajima, 1981; Wright, 1931). Our simulations of the founder events show that the proportions of outlier loci we detect in both introductions are comparable to neutral expectations (Table 3). The simulations do not account for some of the biases associated with RADseq data such as allelic dropout and missing data, but these differences are unlikely to change these conclusions. The smallest bottleneck size (*N*_e_ = 20) produced a larger proportion of allele frequency differences than our observed values, suggesting less extreme founding events in the House Finch.

Although a moderate bottleneck can explain most or all of the observed differences in allele frequency between native and introduced populations, some shifts may have been driven by selection in the novel environments experienced by the introduced populations. The eastern population has smaller legs and feet than the western population (Aldrich & Weske, 1978), and males and females of the eastern population exhibit heritable, sex‐specific patterns of covariance among mensural characters (Badyaev & Hill, 2000). The eastern population also exhibits significantly more short distance migration (Able & Belthoff, 1998) and, possibly as a consequence, has more pointed wings (Egbert & Belthoff, 2003). The Hawaiian population has a smaller body size and greater morphological differentiation compared to the eastern population (Vazquez‐Phillips, 1992). These morphological and behavioral changes likely have at least a partial genetic basis, and selection for these phenotypic traits may be responsible for some of the changes in allele frequencies. With our dataset, we cannot compare an empirical distribution of outlier loci with loci known a priori to be evolving neutrally (Lotterhos & Whitlock, 2014). But, BayeScan has been used to detect selection in populations that have undergone bottlenecks (e.g., Pilot et al., 2013). Of the 224 outliers we documented in the Pre‐E population relative to Pre‐W, and the 125 outliers in the Hawaiian population relative to Pre‐W, we found enrichment for several gene ontology terms, but none with obvious implications for observed phenotypic differences (Supplement methods and results; Table S5). Although BayeScan has low power if few populations are compared (De Mita et al., 2013), it detected 2–4 outliers in each comparison. Of the SNPs that could be mapped to the Zebra Finch genome, all were in intergenic regions, and the closest genes had unknown functions (Tables S3 and S4). As Domingues et al. (2012) demonstrated with a founder event 3,000 years ago in beach mice (*Peromyscus*), we find that signatures of selection are likely obscured in founder events on historical time scales due to genetic drift.

### 4.3 Signatures of selection as a result of the epizootic

Museum collections provide important historical snapshots and the opportunity to study changes in genetic diversity directly (e.g., Therkildsen et al., 2013; Tin et al., 2015). More importantly, these collections allow us to quantify the effects of anthropogenic change in wild organisms (Bi et al., 2013; Foster et al., 2007; Habel, Husemann, Finger, Danley, & Zachos, 2013; Leonard, 2008; Nielsen & Hansen, 2008; Wandeler, Hoeck, & Keller, 2007). Despite substantial decreases in census size after the MG epizootic (Nolan et al., 1998), we found no genome‐wide signatures of a temporal bottleneck induced by the epizootic in eastern House Finches, suggesting that the effective population size remained stable despite substantial epizootic‐driven decreases in census population sizes. Such a pattern is perhaps expected, given that there were still millions of individuals in the population, and genetic drift would likely have minimal effects. However, with such a large population size, we expect some signatures of selection in this system between Pre‐ and Post‐E birds. This expectation is further strengthened given the demonstrated increase in resistance (Bonneaud et al., 2011, 2012) in experimentally infected birds and decreases in bill length, tarsus length, and wing chord (Nolan et al., 1998) exhibited by post‐epizootic as compared to pre‐epizootic birds in the field. Our inability to detect significant temporal shifts in allele frequency in the eastern United States may be expected given the low levels of LD in the population, the sparse sampling of the House Finch genome by ddRADseq (one locus approximately every 5 MB), and what was likely a mild selection event even in the face of substantial drops in census numbers. Even so, our Post‐E sample size was higher than that for Hawaii, where we easily found a strong evidence for allele frequency shifts among islands and relative to the western United States.

The values of Tajima's *D* in pre‐ and post‐epizootic populations were similar and nonsignificant across space and time, suggesting that none of the populations departed significantly from a neutral model. The exception was for the post‐epizootic New York population, which also had the smallest sample size possible for calculating Tajima's *D* (four individuals). Small sample size has been shown to inflate values of Tajima's *D* (Subramanian, 2016). Overall, these results suggest that there is little genome‐wide deviation from a neutral model in House Finches.

To detect loci associated with the previously documented temporal and geographic differences in gene expression exhibited by House Finches in experimental infections with MG (Bonneaud et al., 2011), we likely need denser sampling of the House Finch genome (Andrews et al., 2016; Edwards et al., 2016; Tiffin & Ross‐Ibarra, 2014). More sensitive methods for detecting selection using both polymorphism and LD require extended tracts of sequence in genomic regions surrounding selected loci (Barrett & Schluter, 2008; Sabeti, 2006; Vitti, Grossman, & Sabeti, 2013). Studies seeking to quantify the effects of selection events on genomes in natural populations with large effective population sizes such as the House Finch likely need to employ whole genome resequencing to maximize sampling throughout the genome.

## 5 Conclusions

In summary, by using both temporal and geographic sampling of House Finch populations, we extend the previous findings, suggesting signatures of human‐induced founder events on both the eastern United States and Hawaiian populations of House Finch, with signatures of reduced heterozygosity, reduced genetic diversity, reduced haplotype diversity, and increased LD at very short genomic distances (within 140 base pairs). We detected loci with shifted allele frequencies as a consequence of the human‐induced founder events and suggest that these shifts are likely more often caused by genetic drift than selection. Despite a favorable scenario for detecting pathogen‐mediated signatures of selection in the eastern United States with temporal sampling (no bottleneck, putatively strong selection, known phenotypic differences), RADseq was unable to detect genome‐wide reductions in diversity or loci with significantly different allele frequencies before and after the epizootic, results likely driven by overall low levels of LD throughout the House Finch genome, as well as the low density of markers generated by ddRADseq. Overall, our study provides a rare direct comparison of temporal and spatial events within the same species and confirms a hypothesis that is rarely tested in side‐by‐side comparisons: That demographic shifts, such as bottlenecks or range expansions, may have more profound and genome‐wide consequences for genomic variation than will selection imposed by a novel pathogen. Therefore, our study suggests that, despite conservation concern with selective events such as epizootics, if populations maintain sufficient effective population sizes to mitigate the effects of genetic drift, there may be few genomic consequences of such events in nature.

## Funding Information

## Conflict of Interest

None declared.

## Data Deposition

Data available from the Dryad Digital Repository: http://dx.doi.org/10.5061/dryad.0h2g0.
