# Supplementary material for: SNPs across time and space: population genomic signatures of founder events and epizootics in the House Finch (Haemorhous mexicanus)
Source: Ecol Evol. 2016 Sep 28;6(20):7475–89. doi: 10.1002/ece3.2444 (PMC5513257; doi:10.1002/ece3.2444)
Supplement: Supplementary file 1 [file ECE3-6-7475-s001.pdf]

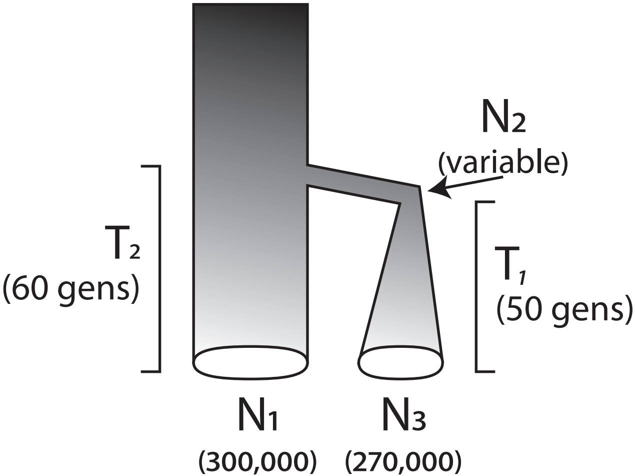

Supplemental Figure 1: The model used in ms simulations

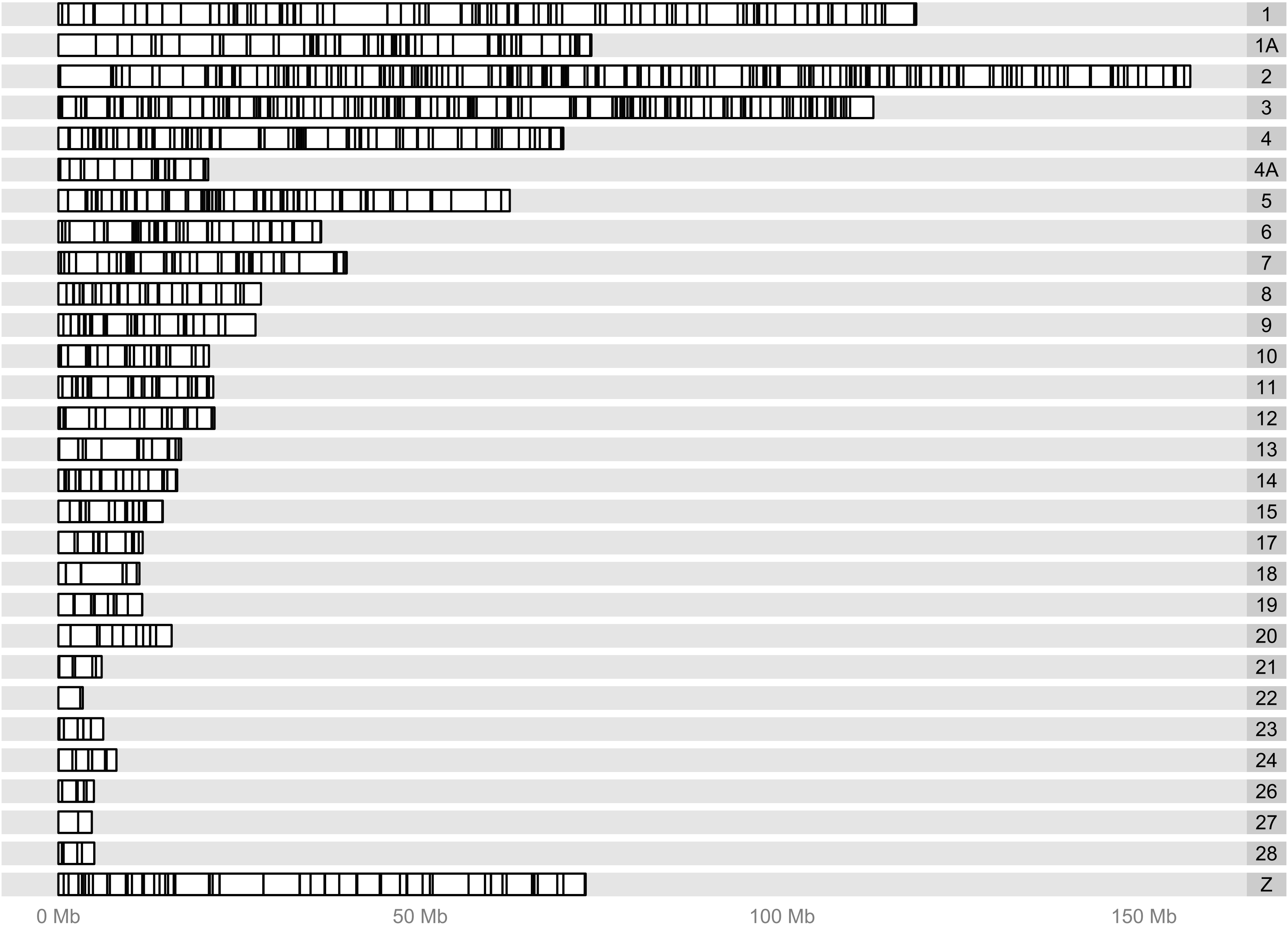

Supplemental Figure 2: Ideogram of the locations of all loci from dataset 1 (minimum locus depth of 10) mapped to the Zebra Finch genome. Each horizontal bar represents the chromosomes, scaled by its length. Each vertical black line represents the position along the chromosome of one of the loci.

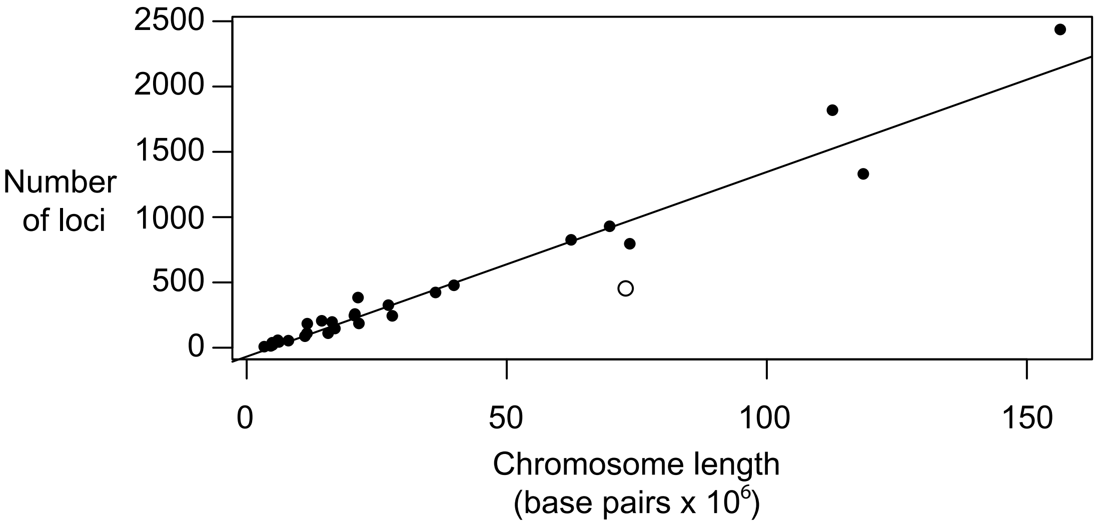

Supplemental Figure 3: The number of loci per chromosome as a function of chromosome length for dataset 1. Each point represents a single chromosome, and the white point shows the Z chromosome. Note that although only dataset 1 is depicted, it is representative of all three datasets. The Z chromosome exhibited fewer loci than expected given its length, possibly due to its smaller effective population size, increased locus dropout due to decreased coverage in females, or increased divergence between the Zebra Finch and House Finch due to the "Faster-Z" effect (Mank et al. 2007) resulting in decreased mapping.

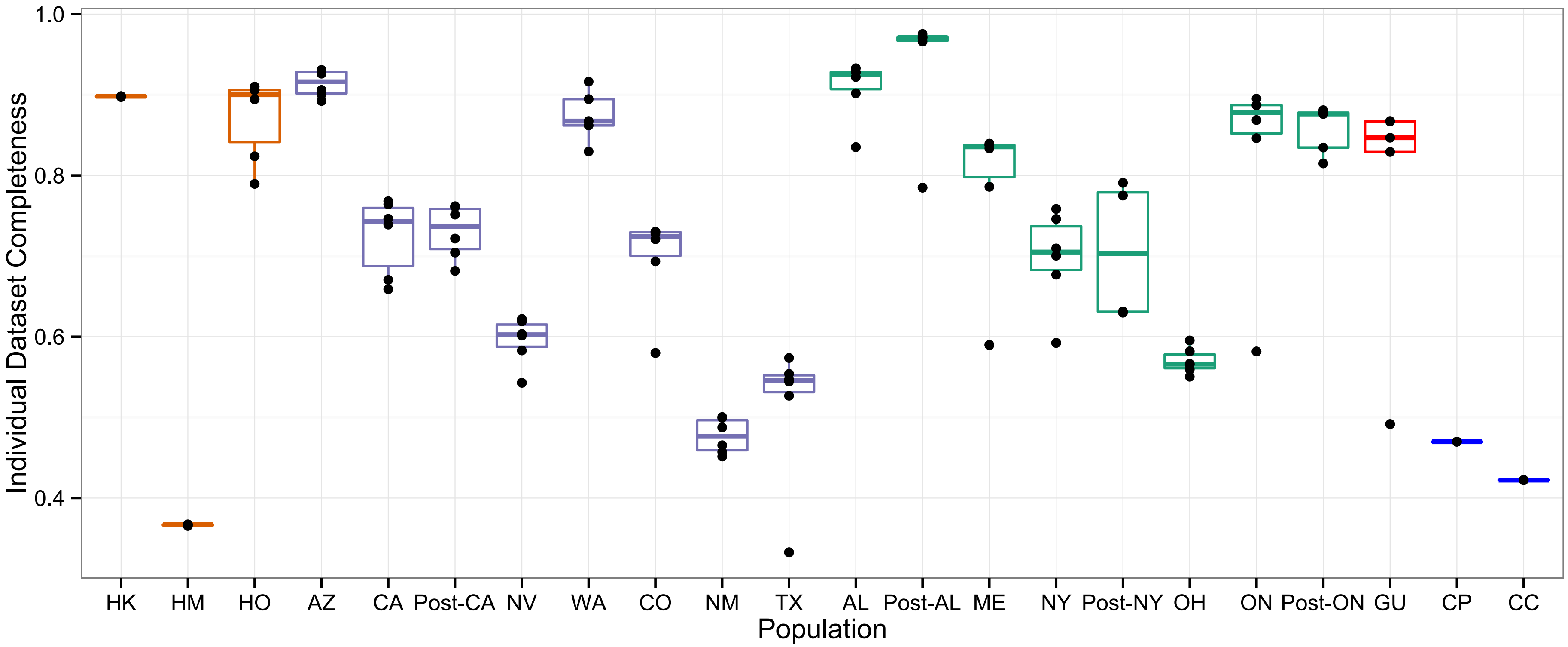

Supplemental Figure 4: For dataset 1, individual dataset completeness, subdivided by subpopulation and colored by population (green = eastern, purple = western, orange = Hawaiian, blue = species outgroup, red = griscomi subspecies). All populations refer to samples collected in the first time period before MG unless labeled as "Post", indicating samples collected in the second time period, after MG or the equivalent time in the western population.

		Western							Eastern					Hawaii			Outgroups		
		AZ	CA	CO	NM	NV	TX	WA	AL	ME	NY	OH	ON	HK	HM	HO	GU	CC	CP
Western	AZ		0.054	0.054	0.055	0.057	0.053	0.059	0.062	0.062	0.063	0.061	0.064	0.086	0.077	0.072	0.128	0.414	0.422
	CA	0.053		0.056	0.056	0.058	0.056	0.060	0.061	0.061	0.062	0.062	0.062	0.087	0.083	0.071	0.137	0.433	0.439
	CO	0.054	0.056		0.056	0.057	0.056	0.062	0.063	0.065	0.065	0.064	0.067	0.091	0.081	0.072	0.135	0.426	0.428
	NM	0.055	0.054	0.054		0.055	0.057	0.063	0.066	0.067	0.068	0.064	0.068	0.091	0.083	0.077	0.133	0.404	0.416
	NV	0.054	0.053	0.056	0.053		0.058	0.064	0.063	0.066	0.065	0.065	0.068	0.092	0.080	0.078	0.137	0.410	0.410
	TX	0.052	0.056	0.057	0.055	0.054		0.063	0.064	0.066	0.068	0.066	0.070	0.091	0.084	0.077	0.139	0.428	0.434
	WA	0.058	0.056	0.061	0.059	0.061	0.062		0.069	0.069	0.070	0.069	0.070	0.102	0.093	0.079	0.149	0.457	0.464
Eastern	AL	0.062	0.060	0.067	0.067	0.063	0.068	0.068		0.053	0.054	0.049	0.053	0.098	0.089	0.082	0.148	0.446	0.451
	ME	0.060	0.059	0.068	0.067	0.062	0.065	0.066	0.055		0.053	0.053	0.054	0.099	0.089	0.081	0.149	0.450	0.457
	NY	0.063	0.060	0.068	0.066	0.065	0.069	0.070	0.054	0.053		0.054	0.055	0.102	0.093	0.084	0.153	0.454	0.457
	OH	0.060	0.057	0.064	0.065	0.062	0.064	0.068	0.050	0.052	0.053		0.052	0.098	0.089	0.081	0.146	0.443	0.446
	ON	0.064	0.063	0.069	0.068	0.066	0.068	0.070	0.056	0.055	0.056	0.051		0.102	0.093	0.085	0.155	0.454	0.465
Hawaii	HK	0.088	0.089	0.092	0.091	0.089	0.092	0.103	0.108	0.098	0.102	0.096	0.107		0.129	0.099	0.213	0.542	0.555
	HM	0.084	0.092	0.099	0.087	0.118	0.092	0.100	0.106	0.091	0.110	0.100	0.086	0.163		0.076	0.184	0.519	0.538
	HO	0.073	0.067	0.072	0.080	0.076	0.077	0.076	0.082	0.082	0.086	0.080	0.089	0.099	0.064		0.166	0.482	0.492
Out	GU	0.128	0.131	0.138	0.125	0.134	0.135	0.150	0.157	0.153	0.149	0.150	0.161	0.217	0.175	0.175		0.524	0.535
	CC	0.416	0.435	0.431	0.392	0.409	0.412	0.453	0.461	0.450	0.448	0.421	0.461	0.541	0.547	0.489	0.524		0.349
	CP	0.418	0.425	0.434	0.416	0.411	0.431	0.450	0.457	0.447	0.448	0.432	0.464	0.550	0.586	0.486	0.535	0.325	

0.586

0.049

Supplemental Figure 5: Pairwise FST estimates of genetic distance among all sampled subpopulations as calculated by STACKS. The results from dataset 1 (minimum locus depth of 10) are in the upper triangle, and the results from dataset 2 (minimum locus depth of 30) are in the bottom triangle. Each comparison is colored by the FST value, ranging from blue for the lowest values to red for the highest values.

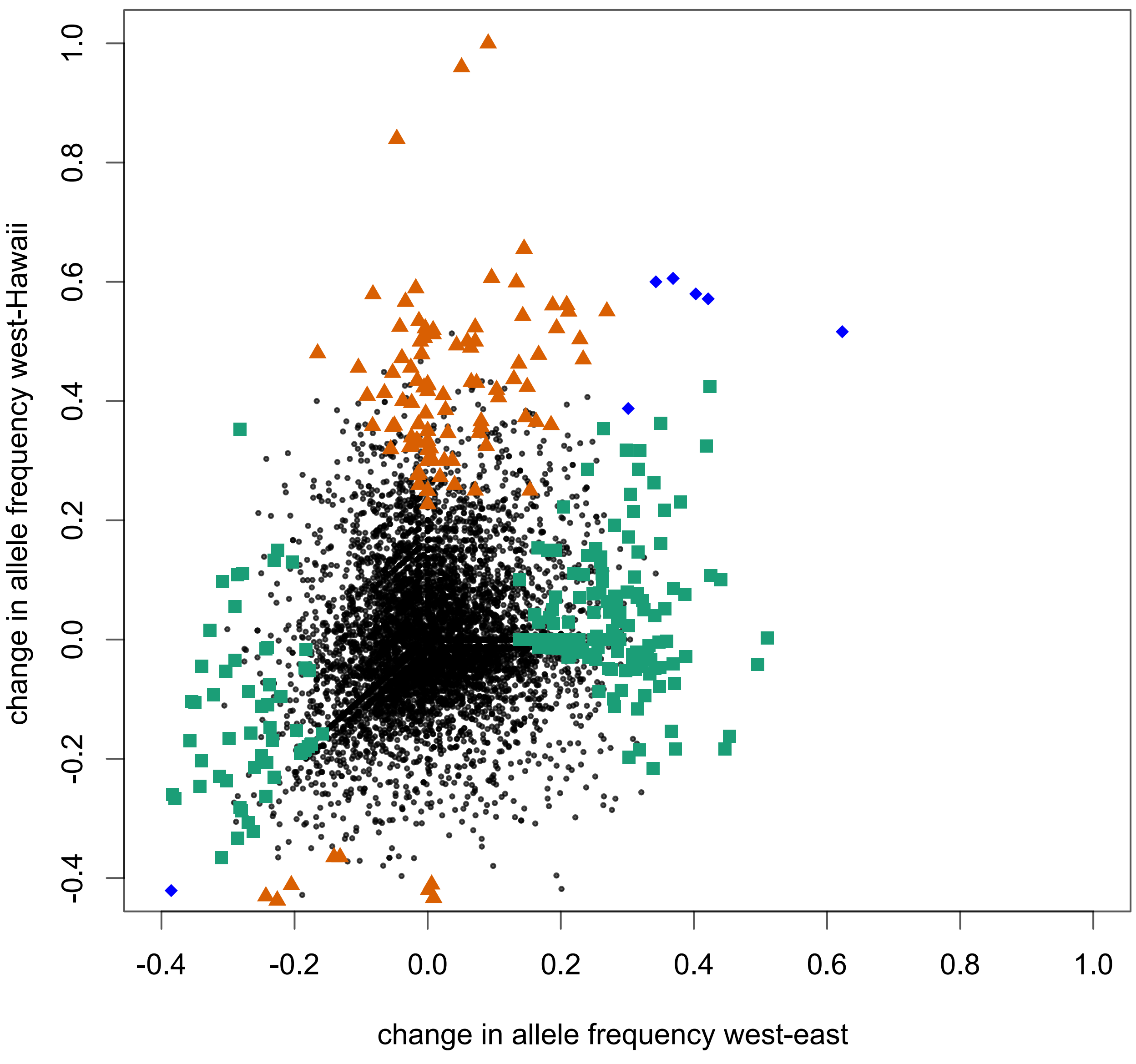

Supplemental Figure 6: Comparison of the major allele frequencies between the Pre-W and Pre-E populations (significant Fisher's exact test outlier loci shown as green squares), and the Pre-W and Hawaiian populations (significant Fisher's exact test outlier loci shown as orange triangles). Loci that are significant in both comparisons are depicted as blue diamonds.

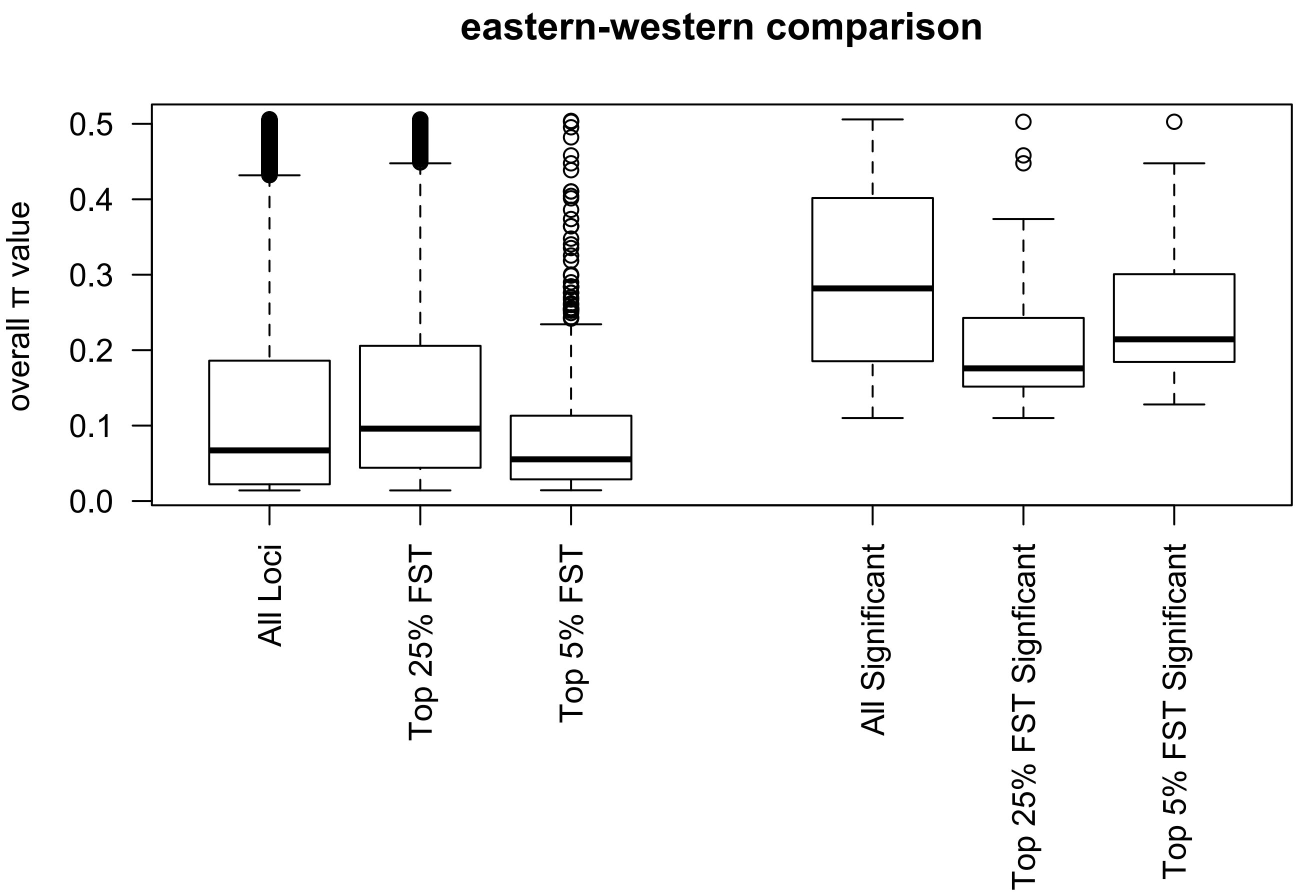

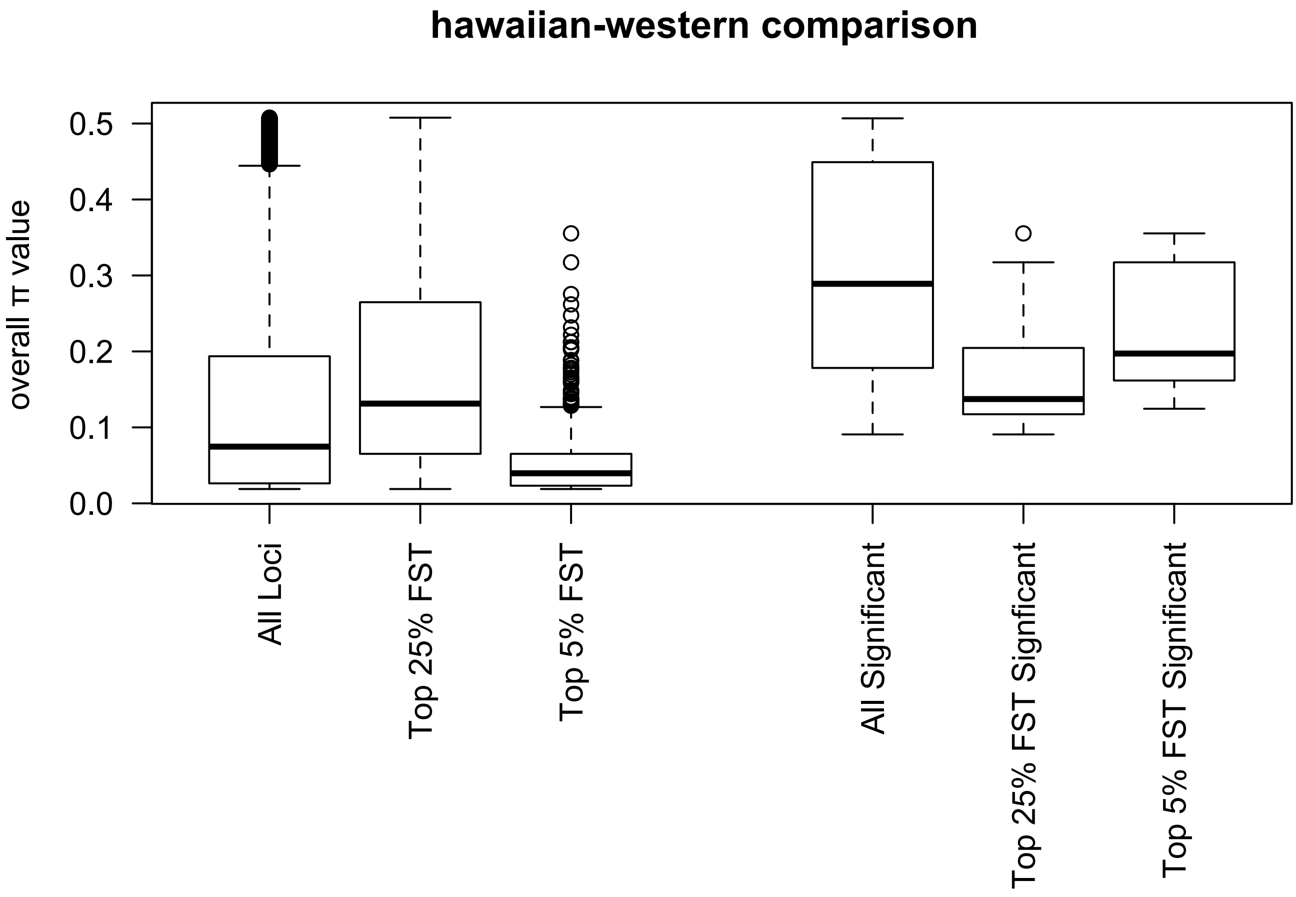

Supplemental Figure 7: Overall estimates of π for all SNPs versus outlier SNPs for Pre-E and Pre-W comparisons and Hawaiian and Pre-W comparisons.

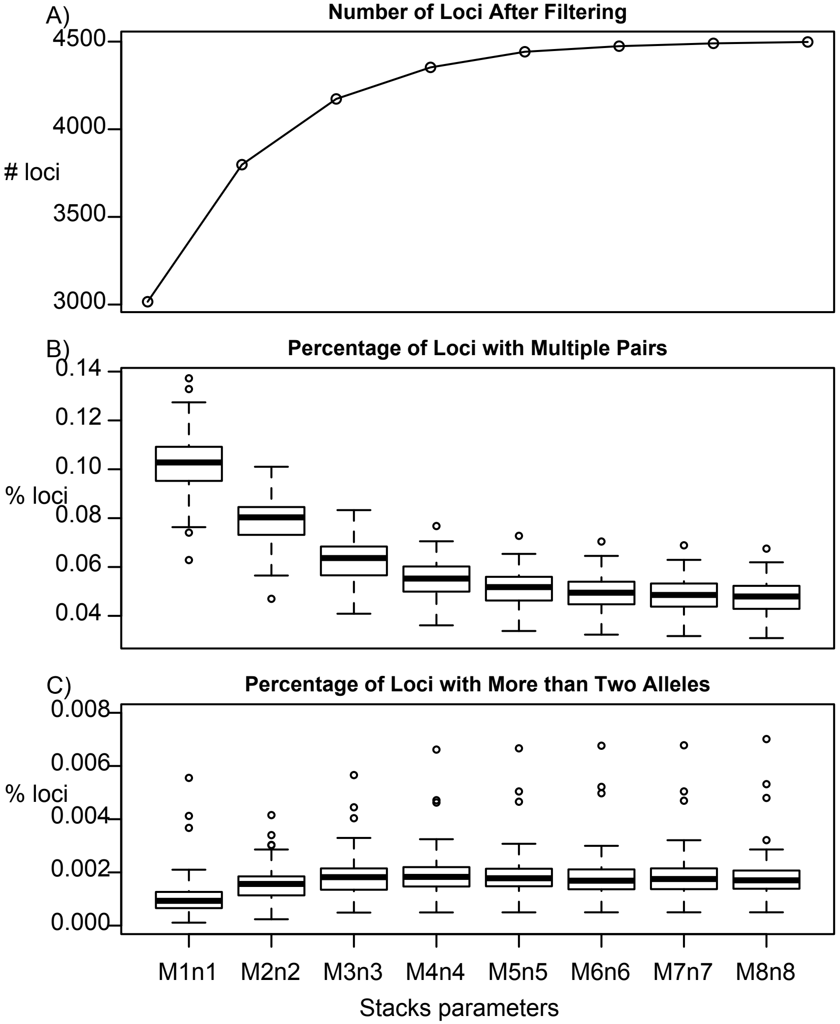

Supplemental Figure 8: A comparison of different –M and –n parameters in denovo_map.pl to build the STACKS catalog. A) The number of loci left after using the populations program with the following filters: an individual must have a minimum stack depth of 10 to be included (-m 10), and a locus may only be included if it was present in at least 75% of individuals in half of the populations (-r 0.75 and –p 9). B) The percentage of loci paired from the same DNA fragment that had more than a one-to-one match within an individual. We show a boxplot summarizing results across all individuals in the catalog. C) The percentage of loci with more than two alleles within an individual, shown with a boxplot summarizing results across all individuals in the catalog.

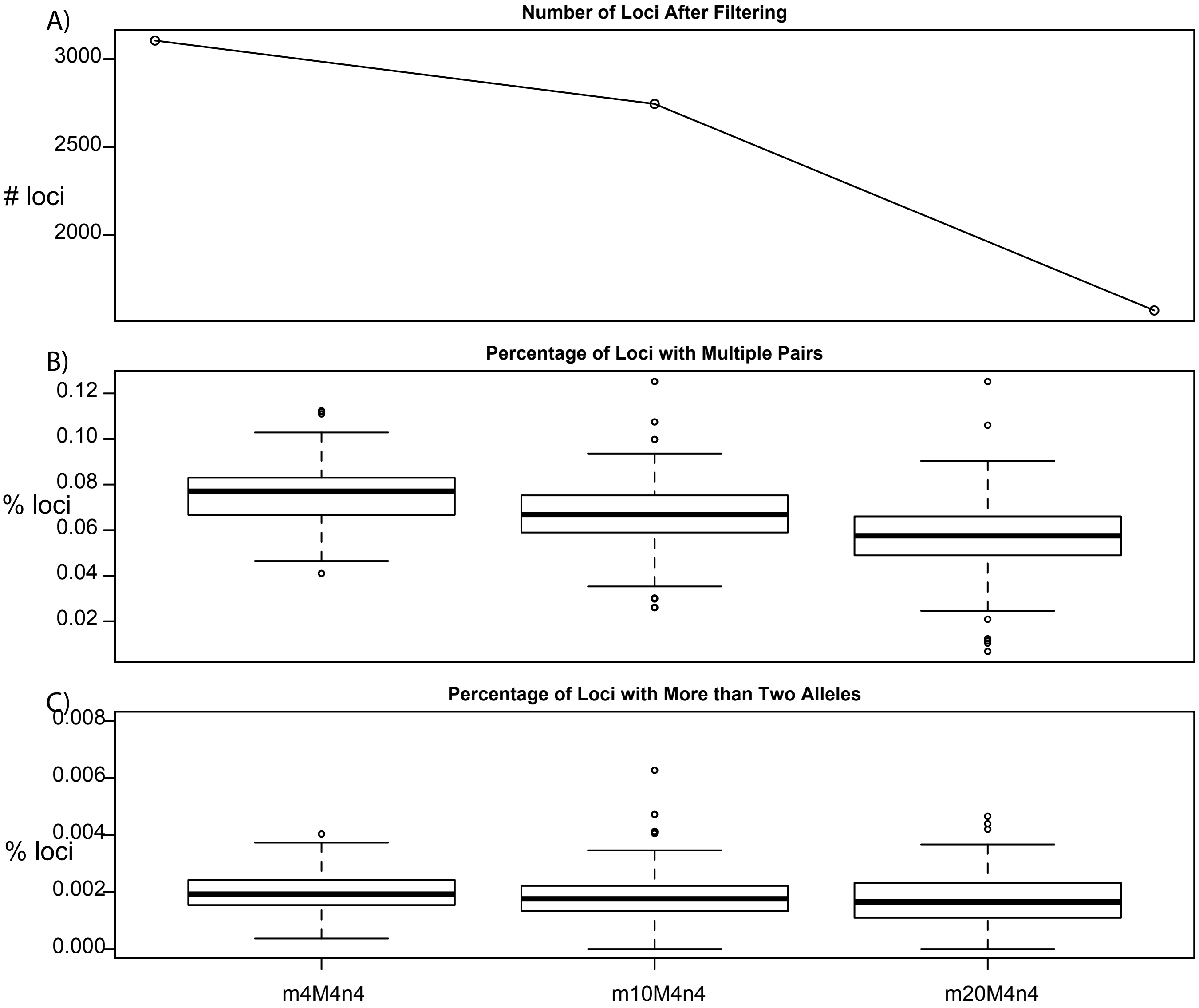

Supplemental Figure 9: Comparing different –m parameters in denovo_map.pl to build the STACKS catalog. A) The number of loci left after using the populations program with the following filters: an individual must have a minimum stack depth of 10 to be included (-m 10), and a locus may only be included if it was present in at least 75% of individuals in half of the populations (-r 0.75 and –p 9). B) The percentage of loci paired from the same DNA fragment that had more than a one-to-one match within an individual. We show a boxplot summarizing results across all individuals in the catalog. C) The percentage of loci with more than two alleles within an individual, shown with a boxplot summarizing results across all individuals in the catalog.
