# Supplementary material for: SNPs across time and space: population genomic signatures of founder events and epizootics in the House Finch (Haemorhous mexicanus)
Source: Ecol Evol. 2016 Sep 28;6(20):7475–89. doi: 10.1002/ece3.2444 (PMC5513257; doi:10.1002/ece3.2444)
Supplement: Supplementary file 7 [file ECE3-6-7475-s007.docx]

**Supplemental Table 6.**

| **Species** | **Scientific Name** | **π** | **Citation** |
| --- | --- | --- | --- |
| Collared Flycatcher | *Ficedula albicollis* | 0.00370 | Ellegren et al. 2012 |
| Three-spined Stickleback | *Gasterosteus aculeatus* | 0.00336 | Hohenlohe et al. 2010 |
| European eel | *Anguilla anguilla* | 0.00529 | Pujolar et al. 2013 |
| Bumblebee | *Bombus impatiens* | 0.00410 | Lozier 2014 |
| Bumblebee | *Bombus pensylvanicus* | 0.00440 | Lozier 2014 |
| Swainson's Thrush | *Catharus ustulatus ustulatus* | 0.00140 | Ruegg et al. 2014 |
| Swainson's Thrush | *Catharus ustulatus swainsoni* | 0.00180 | Ruegg et al. 2014 |
| Zebra Finch (mainland) | *Taeniopygia guttata* | 0.01100 | Balakrishnan and Edwards 2008 |
| Black-footed Albatross | *Phoebastria nigriceps* | 0.00060 | Dierickx et al. 2015 |

**References:**

Balakrishnan, C. N., and S. V. Edwards. 2008. Nucleotide variation, linkage disequilibrium and founder-facilitated speciation in wild populations of the Zebra Finch (*Taeniopygia guttata*). Genetics 181:645–660.

Dierickx, E. G., A. J. Shultz, F. Sato, T. Hiraoka, and S. V. Edwards. 2015. Morphological and genomic comparisons of Hawaiian and Japanese Black‐footed Albatrosses (Phoebastria nigripes) using double digest RADseq: implications for conservation. Evol Appl 8:662–678.

Ellegren, H., L. Smeds, R. Burri, P. I. Olason, N. Backström, T. Kawakami, A. Kunstner, H. Mäkinen, K. Nadachowska-Brzyska, A. Qvarnström, S. Uebbing, and J. B. W. Wolf. 2012. The genomic landscape of species divergence in *Ficedula* flycatchers. Nature 491:756-760.

Hohenlohe, P. A., S. Bassham, P. D. Etter, N. Stiffler, E. A. Johnson, and W. A. Cresko. 2010. Population genomics of parallel adaptation in Threespine Stickleback using sequenced RAD tags. PLoS Genet 6:e1000862.

Lozier, J. D. 2014. Revisiting comparisons of genetic diversity in stable and declining species: assessing genome-wide polymorphism in North American bumble bees using RAD sequencing. Mol. Ecol. 23:788–801.

Pujolar, J. M., M. W. Jacobsen, J. Frydenberg, T. D. Als, P. F. Larsen, G. E. Maes, L. Zane, J. B. Jian, L. Cheng, and M. M. Hansen. 2013. A resource of genome-wide single-nucleotide polymorphisms generated by RAD tag sequencing in the critically endangered European eel. Mol. Ecol. Res. 13:706–714.

Ruegg, K., E. C. Anderson, J. Boone, J. Pouls, and T. B. Smith. 2014. A role for migration-linked genes and genomic islands in divergence of a songbird. Mol. Ecol. 23:4757-4769.
