# Supplementary material for: SNPs across time and space: population genomic signatures of founder events and epizootics in the House Finch (Haemorhous mexicanus)
Source: Ecol Evol. 2016 Sep 28;6(20):7475–89. doi: 10.1002/ece3.2444 (PMC5513257; doi:10.1002/ece3.2444)
Supplement: Supplementary file 8 [file ECE3-6-7475-s008.pdf]

Supplemental Methods

Library Preparation. Following the protocol of (Peterson et al. 2012), we digested 500ng to 1000ng of DNA from each individual for three hours at 37°C with SphI and EcoR1. Following an Ampure XP bead purification step (Beckman-Coulter, Fullerton, CA), we ligated one of ten unique P1 "flex" adapter 5bp barcoded oligos and a universal biotin-labeled P2 oligo, and pooled each set of ten uniquely labeled individuals together in equimolar amounts following quantification with a Qubit fluorometer (Invitrogen Corp, Carlsbad, CA). We cleaned each pool with an additional Ampure XP bead purification, and used a Pippin Prep instrument (Sage Sciences, Beverly, MA) to isolate fragments in the range of 345-407 base pairs (bp). We then removed ligation products with the P1 adapter on both ends using Streptavidin-coated Dynabeads. We amplified each pool with 16 cycles of high fidelity KAPA Long Range PCR (KAPA Biosystems, Wilmington, MA) using a universal PCR1 primer and uniquely 6bp barcoded PCR2 primer. We performed an additional Ampure XP bead clean up, and combined each pool into a single library in equimolar amounts. We quantified the molarity of our final library using a Qubit fluorometer and qPCR (KAPA Library Quantification Kit, KAPA Biosystems, Wilmington, MA), and the fragment size distribution using a BioAnalyzer (Agilent Technologies, Santa Clara, CA).

De novo assembly parameter testing. We tested several different parameters to choose the best combination for this dataset. First, we varied the -M parameter, which specifies the number of allowed nucleotide mismatches within an individual to group similar stacks of identical reads into a locus. We varied -M from one to eight, keeping the

minimum number of reads required to form a stack (-m) constant at four, and the number of allowed nucleotide mismatches among locus stacks among individuals to form loci in the library, -n, the same as the -M parameter. We then quantified the total loci in the catalog after running the *populations* program from STACKS with the following filters: an individual must have a minimum locus depth of 10 to be included (-m 10), and a locus may only be included if it was present in at least 75% of individuals in half of the populations (-r 0.75 and –p 9). We then assessed the effects of changing these parameters with three different metrics using a series of custom python scripts (found at *https://github.com/ajshultz/Rad/*). First, we leveraged our paired-end sequencing to quantify the number of loci sequenced from read 1 and read 2 of the same fragment that paired together with single loci (e.g. a stack that forms one locus always paired with a single other locus when mapped to physical DNA fragments). The novel script *indiv_read_pair_module.py* performs this calculation for each locus within an individual, and *catalog_read_pair.py* performs these calculations for each individual, integrating the results across all individuals on a per-locus basis. Next, we used the novel script *excess_allele_calc.py* to calculate the number of loci for which any individuals possessed more than two alleles. Such a result can arise for two reasons. First, because of errors in a small number of reads that would normally be corrected in Stacks. However, if these errors occur in a SNP site, they are not discarded and cause the phasing to appear incorrect and a false haplotype to be called. Second, because paralogous and repeat loci are incorrectly collapsing into a single locus.

We found that as –M and –n increased, the estimated number of loci increased rapidly and then plateaued at about 4,500 loci (Supplemental Figure 9A), the percentage

of the total loci per individual that had more than one locus in a pair decreased and then plateaued (Supplemental Figure 9B), and the percentage of loci with more than two alleles within an individual increased and then plateaued (Supplemental Figure 9C). The increasing number of loci and decreasing percentage of loci with multiple pairs suggest that as –M and –n increases, polymorphic loci are correctly collapsing into more complete datasets. The increasing percentage of loci with more than two alleles suggests that there might be some paralogous loci that are incorrectly collapsing, but the overall proportion is small. For downstream analyses, we chose the –M 4 and –n 4 parameters, because after this parameter combination the curves appeared to asymptote. We experimented with increasing –n because there may be additional polymorphisms in a locus among individuals, but these had little effect on the final catalog, similar to the findings of (Mastretta-Yanes et al. 2014). We also tested the effects of increasing the –m parameter to 10 and 20, but found that increasing -m caused many loci to drop out of the analysis, but only had a small effect on the other metrics described above (Supplemental Figure 10), so we decided to use –m 4 to build our library and filter more conservatively for overall locus depth further down the pipeline using the *populations* program.

ms Modeling. Commands used in the program ms (Hudson 2002) to generate simulated data for the eastern population comparison (the same commands were used with Hawaii, but with appropriate sample sizes (-I 2 82 24).

Bottleneck size of 20: ms 142 1000 -t 795 -I 2 82 60 -n 1 1 -n 2 0.80 -g 2 228068 -eg 0.0000417 2 0.0 -en 0.00005 2 0.0000667 -ej 0.00005 2 1 > ms_out_bottle20_EW.txt

Bottleneck size of 200: ms 142 1000 -t 795 -I 2 82 60 -n 1 1 -n 2 0.90 -g 2 172850 -eg 0.0000417 2 0.0 -en 0.00005 2 0.000667 -ej 0.00005 2 1 > ms_out_bottle209_EW.txt

Bottleneck size of 2,000: ms 142 1000 -t 795 -I 2 82 60 -n 1 1 -n 2 0.80 -g 2 117632 -eg 0.0000417 2 0.0 -en 0.00005 2 0.00667 -ej 0.00005 2 1 > ms_out_bottle2000_EW.txt

Bottleneck size of 100,000: ms 142 1000 -t 795 -I 2 82 60 -n 1 1 -n 2 0.80 -g 2 23818 -eg 0.0000417 2 0.0 -en 0.00005 2 0.33 -ej 0.00005 2 1 > ms_out_bottle100000_EW.txt

No bottleneck: ms 142 1000 -t 795 -I 2 82 60 -n 1 1 -n 2 1 -ej 0.00005 2 1 > ms_out_nobottle_EW.txt

GO Term Enrichment: We annotated all SNPs using the VariantAnnotation package version 1.12.9 (Obenchain et al. 2014) within Bioconductor version 3.0 (Gentleman et al. 2004) for all loci that mapped to the Zebra Finch genome using the Zebra Finch gene positions and gene ontology categories downloaded from the ensembl dataset via the biomaRt package (Durinck et al. 2005; 2009). We annotated all SNPs Intergenic variants were associated with the closest gene within 100kb, and variants more than 100kb from any gene were considered "unassigned". We tested for functional enrichment in sets of genes significantly different in populations using TopGO version 2.18.0 (Alexa and Rahnenfuhrer n.d.). We used TopGO to compare the differences in the number of loci associated with a given functional category (gene ontology category, or GO term) between the outlier loci set and set of all genes associated with variants in the entire dataset, and assessed significance using a Fisher's exact test. In addition to the "classic" algorithm, which only considers raw count data for a given GO term, we also calculated scores for the "elim" algorithm, which considers the underlying GO graph topology from a bottom-up approach, the "weight" algorithm, which weights genes according to the significance of its neighboring nodes, and the "weight01" algorithm, which is a combination of all three scores and has a low false-positive rate (Alexa et al. 2006).

Supplemental Results

GO Term Enrichment: Across the entire dataset, 839 genes fell within 100kb of the nearest ddRADseq SNP; this set was used as the background set of possible genes for the topGO analysis. For the Pre-E/W comparison, 65 of the 136 mappable SNPs showing significant differences between populations were within 100kb of a gene, and were assigned to the closest gene. Of these, there were 53 unique genes used in the topGO analysis, which resulted in five GO terms with significant "weight01" p-values ($p < 0.05$) and more than one gene annotated (Supplemental Table 5). For the Hawaiian vs. Pre-W comparison, 36 of the 84 highly differentiated and mappable SNPs were within 100kb of and assigned to the closest gene, yielding 31 unique genes and resulting in ten signficant GO terms (Supplemental Table 5).

Literature Cited:

Alexa, A., and J. Rahnenfuhrer. n.d. topGo: Enrichment analysis for Gene Ontology.

Alexa, A., J. Rahnenfuhrer, and T. Lengauer. 2006. Improved scoring of functional groups from gene expression data by decorrelating GO graph structure. Bioinformatics (Oxford, England) 22:1600–1607.

Durinck, S., P. T. Spellman, E. Birney, and W. Huber. 2009. Mapping identifiers for the integration of genomic datasets with the R/Bioconductor package biomaRt. Nat Protoc 4:1184–1191.

Durinck, S., Y. Moreau, A. Kasprzyk, S. Davis, B. De Moor, A. Brazma, and W. Huber.

2005. BioMart and Bioconductor: a powerful link between biological databases and microarray data analysis. Bioinformatics (Oxford, England) 21:3439–3440.

Gentleman, R. C., V. J. Carey, D. M. Bates, B. Bolstad, M. Dettling, S. Dudoit, B. Ellis, L. Gautier, Y. Ge, J. Gentry, K. Hornik, T. Hothorn, W. Huber, S. Iacus, R. Irizarry, F. Leisch, C. Li, M. Maechler, A. J. Rossini, G. Sawitzki, C. Smith, G. Smyth, L. Tierney, J. Y. H. Yang, and J. Zhang. 2004. Bioconductor: open software development for computational biology and bioinformatics. Genome Biol 5:R80.

Hudson, R. R. 2002. Generating samples under a Wright–Fisher neutral model of genetic variation. Bioinformatics (Oxford, England) 18:337–338. Oxford Univ Press.

Mastretta-Yanes, A., N. Arrigo, N. Alvarez, T. H. Jorgensen, D. Piñero, and B. C. Emerson. 2014. Restriction site-associated DNA sequencing, genotyping error estimation and de novo assembly optimization for population genetic inference. Molecular Ecology Resources 15:28–41.

Obenchain, V., M. Lawrence, V. Carey, S. Gogarten, P. Shannon, and M. Morgan. 2014. VariantAnnotation: a Bioconductor package for exploration and annotation of genetic variants. Bioinformatics (Oxford, England) 30:2076–2078.

Peterson, B. K., J. N. Weber, E. H. Kay, H. S. Fisher, and H. E. Hoekstra. 2012. Double digest RADseq: an inexpensive method for de novo SNP discovery and genotyping in model and non-model species. PLoS ONE 7:e37135.
